# Supplementary material for: Indication of critical scaling in time during the relaxation of an open quantum system
Source: Nat Commun. 2024 Feb 24;15:1714. doi: 10.1038/s41467-024-46054-9 (PMC10894203; doi:10.1038/s41467-024-46054-9)
Supplement: Supplementary file 1 — Supplementary Information [file 41467_2024_46054_MOESM1_ESM.pdf]

Supplementary Information for
Indication of critical scaling in time during the relaxation of an open quantum system

Ling-Na Wu,[1,2] Jens Nettersheim,[3] Julian Feß,[3] Alexander Schnell,[1] Sabrina Burgardt,[3] Silvia Hiebel,[3] Daniel Adam,[3] André Eckardt,[1,*] and Artur Widera[3,†]

[1]*Institut für Theoretische Physik, Technische Universität Berlin, Hardenbergstraße 36, 10623 Berlin, Germany*
[2]*Center for Theoretical Physics and School of Science, Hainan University, Haikou 570228, China*
[3]*Department of Physics and Research Center OPTIMAS,*
University Kaiserslautern-Landau, 67663 Kaiserslautern, Germany
(Dated: February 6, 2024)

In this supplemental material, we delve into the intricacies of our model (see Supplementary Note 1) and provide a comprehensive exploration of additional experimental and numerical data beyond the scope of the main text (see Supplementary Note 2). We also present two approaches to understanding the scaling behavior discussed in the main text (see Supplementary Note 3).

Supplementary Note 1. MODEL

In this section, we elaborate on our model and provide additional details and insights.

A. Model of the experimental realization.

The experimental system comprises individual Cs atoms immersed in a large Rb bath. The Hamiltonian of this Cs-Rb mixture is given by [1]

$$H = E_{\text{coll}} + \sum_{j=\text{Cs},\text{Rb}} (V_j^Z + V_j^{\text{HFS}}) + H_{\text{int}}. \tag{1}$$

Here, E_{coll} denotes kinetic collision energy, V_j^Z and V_j^{HFS} the single-particle Zeeman and hyperfine energies, respectively, and H_{int} describes the interaction of the colliding Rb and Cs atom. The collision energy is well-defined by the relative velocity of the colliding partners for individual collisions, but in the ensemble it is distributed according to a Maxwell-Boltzmann distribution [2]. Considering low collision energies for the experimental ultra-low temperatures , i.e., the s-wave limit, the interaction H_{int} may be efficiently represented in terms of asymptotic Cs (Rb) states, provided by total angular momentum $\mathbf{F}_{\text{Cs}}$ ($\mathbf{F}_{\text{Rb}}$), with quantum numbers F_{Cs} (F_{Rb}) and projection $m_{F,\text{Cs}}$ ($m_{F,\text{Rb}}$) resulting in [3]

$$H_{\text{int}} = \sum_{i=0,1,2} c_i (\mathbf{F}_{\text{Cs}} \cdot \mathbf{F}_{\text{Rb}})^i. \tag{2}$$

In our experiments, Cs (Rb) atoms are in hyperfine ground states $F_{\text{Cs}} = 3$ ($F_{\text{Rb}} = 1$). The interaction energy H_{int} becomes comparable to the hyperfine splitting V_j^{HFS} at distances of few $10a_0$, with a_0 representing the Bohr radius, coupling $\mathbf{F}_{\text{Cs}}$ and $\mathbf{F}_{\text{Rb}}$. The short spatial distance for the interaction justifies an effective contact interaction of the collision. This coupling can lead to different collision channels between Rb and Cs according to

$$|m'_{F,\text{Cs}}, m'_{F,\text{Rb}}\rangle = |m_{F,\text{Cs}} + \Delta m_F, m_{F,\text{Rb}} - \Delta m_F\rangle \tag{3}$$

with $\Delta m_F = 0, \pm 1, \pm 2$. The system collides elastically for $\Delta m_F = 0$, whereas the colliding atoms exchange angular momentum for $\Delta m_F \neq 0$. The latter processes comprise exoergic SE collisions where $\Delta m_F = -1, -2$ and endoergic SE collisions with $\Delta m_F = +1, +2$. Due to the competition between Zeeman and thermal energy (for more details see [4]), endoergic collisions are energetically forbidden for high magnetic fields, leading to a unidirectional spin-exchanging system.

* eckardt@tu-berlin.de
† email: widera@rptu.de

Supplementary Table 1. Experimental parameters of the individual measurements for the unidirectional system, i.e. $B =$ 460(2) mG.

$p_{m_F}(t=0)$	T [nK]	n [10^{13}cm^{-3}]	$\frac{\Gamma_{\mathrm{SE}}}{\Gamma_{\mathrm{elastic}}}$	$\frac{N_{\mathrm{Rb}}}{N_{\mathrm{Cs}}}$	Γ_{SE} [Hz]
$p_0 = 1.00(4)$	900(75)	0.44(9)	0.027	2300	9.69
$p_3 = 0.98(3)$<br>$p_2 = 0.02(1)$	950(67)	0.44(8)	0.026	2595	9.52
$p_2 = 0.90(2)$<br>$p_1 = 0.06(1)$	950(52)	0.49(8)	0.026	2327	10.7
$p_1 = 0.95(2)$<br>$p_0 = 0.05(1)$	900(57)	0.45(6)	0.027	2411	9.96
$p_2 = 0.51(3)$<br>$p_3 = 0.48(2)$	900(52)	0.47(7)	0.027	2708	10.5

Supplementary Table 2. Experimental parameters of the individual measurements for the bidirectional system, i.e. $B =$ 25(2) mG.

$p_{m_F}(0)$	T [nK]	n [10^{13}cm^{-3}]	$\frac{\Gamma_{\mathrm{SE}}}{\Gamma_{\mathrm{elastic}}}$	$\frac{N_{\mathrm{Rb}}}{N_{\mathrm{Cs}}}$	Γ_{SE} [Hz]
$p_1 = 0.66(3)$<br>$p_2 = 0.25(2)$	500(87)	0.41(16)	0.053	7086	22.1
$p_2 = 0.78(3)$<br>$p_1 = 0.11(1)$	450(43)	0.55(12)	0.054	6375	29.5
$p_2 = 0.49(3)$<br>$p_1 = 0.44(2)$	525(55)	0.63(12)	0.053	4591	33.8
$p_0 = 0.85(3)$<br>$p_{-1} = 0.07(1)$	500(47)	0.53(12)	0.053	4844	28.4
$p_{-2} = 0.93(4)$<br>$p_{-3} = 0.06(1)$	525(55)	0.38(11)	0.053	4086	20.5
$p_{-3} = 0.99(4)$<br>$p_{-2} = 0.01(1)$	450(54)	0.58(15)	0.054	3600	31.4

B. Experimental parameters.

Tables 1 and 2 provide the experimental parameters for each data set of the unidirectional and bidirectional model realized at magnetic fields of 460 mG and 25 mG, respectively. The initial population of each data set is illustrated and color-coded as histogram in Fig. 2 of the main text. For simplicity the tables contain the two most populated initial states. The last missing percents of the initial population distribute over the unspecified states in the tables. Table 1 of Methods gives the mean values of the listed temperatures and densities.

C. Markovianity of the experimental bath

In the experimental situation, Rb atoms are prepared in $m_{F,\mathrm{Rb}} = 0$. In this case, angular momentum changes of $\Delta m_F = 0, \pm 1$ are allowed. The number of Rb atoms is approximately three orders of magnitude larger than that

of Cs atoms (see Tables 1 and 2). This strong imbalance between the bath (Rb) and the probe (Cs) together with the physical consequences of the Rb-Cs interaction justifies the assumption of an ideal Markov bath; i.e., Cs atoms exclusively interact with Rb atoms in the internal state ($m_{F,\mathrm{Rb}} = 0$), and correlations by a second collision with the same Rb atom are negligible. First, the rates for elastic collisions are more than a factor 30 larger than SE collisions (see Tables 1 and 2). Additionally, the Cs impurity after an inelastic collision needs approximately three elastic collisions to re-thermalize. Hence, for every SE collision the Cs impurity has the same bath temperature. Furthermore, the total number of atoms produced in state $m_{F,\mathrm{Rb}} \neq 0$ is very small during the interaction time (up to six Rb atoms per Cs impurity). Moreover, the Cs impurity undergoes more than 30 elastic collisions with Rb bath atoms and, furthermore, the mean-free path for our Cs atoms (averaged over all internal states) is larger than $9\,\mu$m ($7\,\mu$m) for the unidirectional (bidirectional) case, while the Rb bath has extensions of $31\,\mu$m ($23\,\mu$m) in axial and $3.2\,\mu$m ($2.4\,\mu$m) in radial direction, respectively. Thus, the Cs impurity samples the whole Rb cloud several times before another SE collision occurs. The probability of colliding with a Rb atom which had previously collided once with the Cs atom is therefore well below the percent level. Hence, experimentally each SE collision occurs with identical conditions involving a Rb atom from an unchanged bath as originally prepared; this scenario realizes the ideal limit of a Markov bath and allows us modelling the time evolution of Cs atoms (driven by SE with Rb) based on a rate equation (see Methods).

Supplementary Note 2. MORE EXPERIMENTAL AND NUMERICAL DATA

In this section, we present a broader range of experimental and numerical data and provide a comprehensive presentation of our results.

A. Role of initial-state energy

Here we discuss the dependence of peak entropy on the initial-state energy. In general, a higher initial energy gives rise to a larger peak entropy. Figure 1 shows the peak entropy in experiments as a function of the initial state energy. One can see that initial states having energies even down to 85% of the maximum initial energy give rise to a peak entropy close (less than 2% distance to S_{max}) to the maximum entropy. In Fig. 2, we show the corresponding results for the theoretical models at much larger system sizes of $M = 100$ and $M = 1000$ states. For both models we find (almost) maximum peak entropies for sufficiently high initial energy. While for the bidirectional model, we find a large basin of initial states giving rise to maximum peak entropies, for the unidirectional model only the most excited states ensure such a large peak entropy (which is a consequence of the fact that the dynamics cannot transport probability upward in energy). However, the latter is sufficient to observe critical behavior in time also for the unidirectional model, as long as the most excited state can approximately be prepared experimentally. Likewise, equilibrium quantum phase transitions happen only in the ground state and not at finite temperature/energy.

B. The mapping between the control parameter β_{eff} and time.

Figure 3 shows the control parameter β_{eff} as a function of time (a,c) and shifted time (b,d) for the unidirectional model (blue background) and the bidirectional model.

C. Finite-size scaling for the unidirectional model with time as control parameter.

It is also possible to directly consider the time t, rather the control parameter β_{eff} as the control parameter. In this case, one has to scale the overall strength of the bath-induced rates with the system size M in such a way that the peak entropy time t_{peak} remains finite in the thermodynamic limit, i.e. that it neither approaches infinity nor zero. For the unidirectional model, we achieve this by scaling the rates like $M^{1.16}$. Note that such overall scaling of the rates, only changes the time scale of the evolution and not the details of the dynamics. (As a consequence, the dynamics plotted with respect to β_{eff} remains unchanged, when choosing a different scaling). Under these conditions the divergence of both ξ and S occurs at (and within) the finite time t_{peak} (see Fig. 4(a)) and, thus, constitutes singular behavior in time like at a continuous phase transition in time. In Fig. 4(b) we present a finite-size scaling analysis of this transition and find that the corresponding critical exponent is given by $1/0.16 = 6.25$. This is consistent with that found in the continuum model (see the discussion in section 'Continuum model').

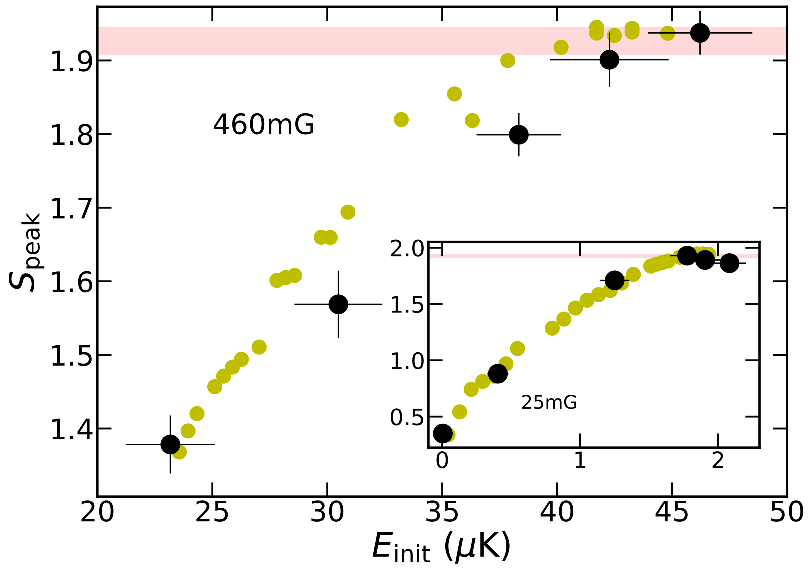

Supplementary Fig. 1. Peak entropy versus initial state energy. The unidirectional and the bidirectional (inset) systems reach both maximum entropy ($S_{\text{peak}} \geq 0.98 S_{\text{max}}$ indicated by the red area) for a broad range of initial energies down to $\approx 85\%$ of the maximum possible energy before the peak entropy decreases. Big black markers illustrate data and small yellow dots simulations. Error bars represent statistical fluctuations of 1σ standard deviation. Simulations consider the same temperature, magnetic field, and atom number, assuming some initial population distributions to sample the initial energy.

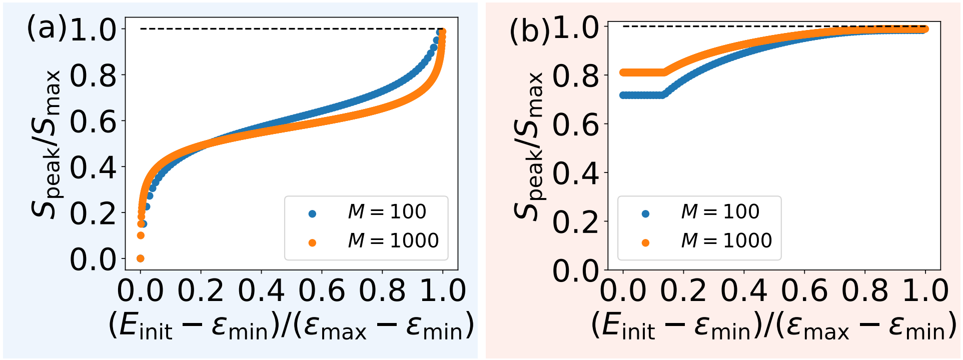

Supplementary Fig. 2. Peak entropy versus initial state energy for the theoretical (a) unidirectional and (b) bidirectional model. The initial states are the eigenstates of the system. The initial energy is scaled between 0, for the energy ϵ_{min} of the ground state, and 1, for the energy ϵ_{max} of the most excited state.

D. Prethermal memory loss.

In the main text, the prethermal memory loss was illustrated for the unidirectional model in Fig. 5. Figure 5 shows the complementary data for the bidirectional model with state-independent rates used in the theoretical model. In Figs. 5(a)-(c), we show the entropy evolution and the population dynamics of two spin states with respect to the shifted time $t - t_{\text{peak}}$. We can see that the data with a high peak entropy ($S_{\text{peak}} \geq 0.98 S_{\text{max}}$, indicated by bullets) show similar behavior for both the entropy evolution and spin dynamics after the system reaches peak entropy ($t - t_{\text{peak}} > 0$). In Fig. 5(d), the difference between trajectories χ_i (see the definition in the main text) is plotted versus control parameter β_{eff}. For those trajectories featuring large peak entropies (indicated by bullets), χ_i becomes small at the transition $\beta_{\text{eff}} = 0$ (though the measured signal is not as clear as for the unidirectional model discussed in the main text), whereas it remains large at $\beta_{\text{eff}} = 0$ for the trajectories with $S_{\text{peak}} < 0.98 S_{\text{max}}$ (indicated by triangles). In Fig. 5(e), we plot normalized χ versus β_{eff} for the theoretical bidirectional model with different system sizes M at $\delta S = 0.02$. One can clearly see that for increasing M, a sharp transition forms at $\beta_{\text{eff}} = 0$.

Prethermal memory loss is also found for both theoretical models, as shown in Fig 6 for systems of size $M = 50$. Panels (a) and (b) show the evolution of the entropy with respect to $t - t_{\text{peak}}$ for various initial conditions (as

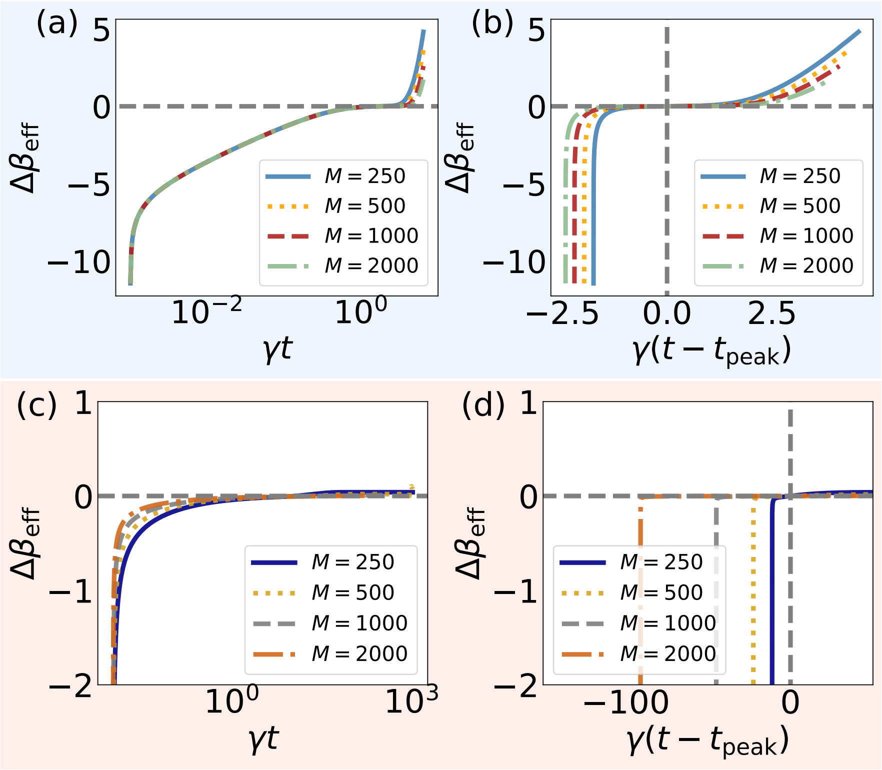

Supplementary Fig. 3. The control parameter $\beta_{\rm eff}$ as a function of time (a,c) or shifted time (b,d) for the unidirectional model (blue background) and the bidirectional model (red background).

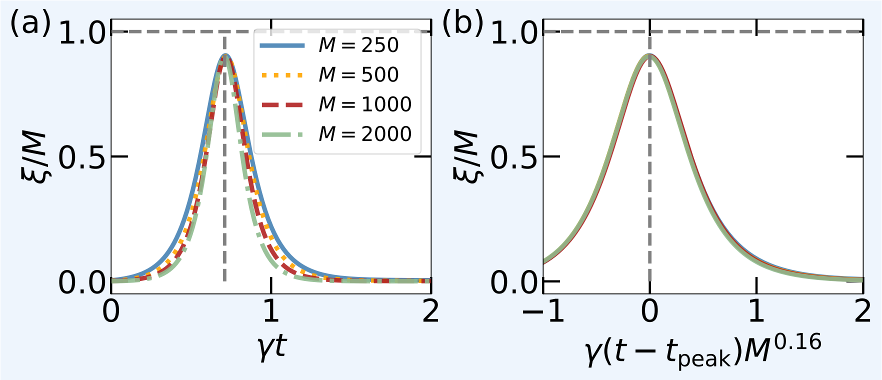

Supplementary Fig. 4. Finite-size scaling for the unidirectional model with time as control parameter. (a) The normalized localization length ξ as a function of time for different system sizes. The overall strength of the bath-induced rates scales with the system size as $M^{1.16}$. The vertical dashed line marks the peak time $t_{\rm peak}$. (b) The normalized localization length ξ as a function of the scaled shifted time (by $t_{\rm peak}$).

indicated by the insets). Panels (c-f) show the corresponding evolution for the populations of two different states for each model. One can clearly observe that (only) for those initial conditions for which the peak entropy closely approaches the maximum possible entropy [dashed line in panels (a) and (b)], the results for different initial conditions converge, when approaching $t = t_{\rm peak}$, to remain very similar at all later times.

Supplementary Note 3. UNDERSTANDING OF THE SCALING BEHAVIOR

In this section, we present two approaches to understanding the scaling behavior discussed in the main text.

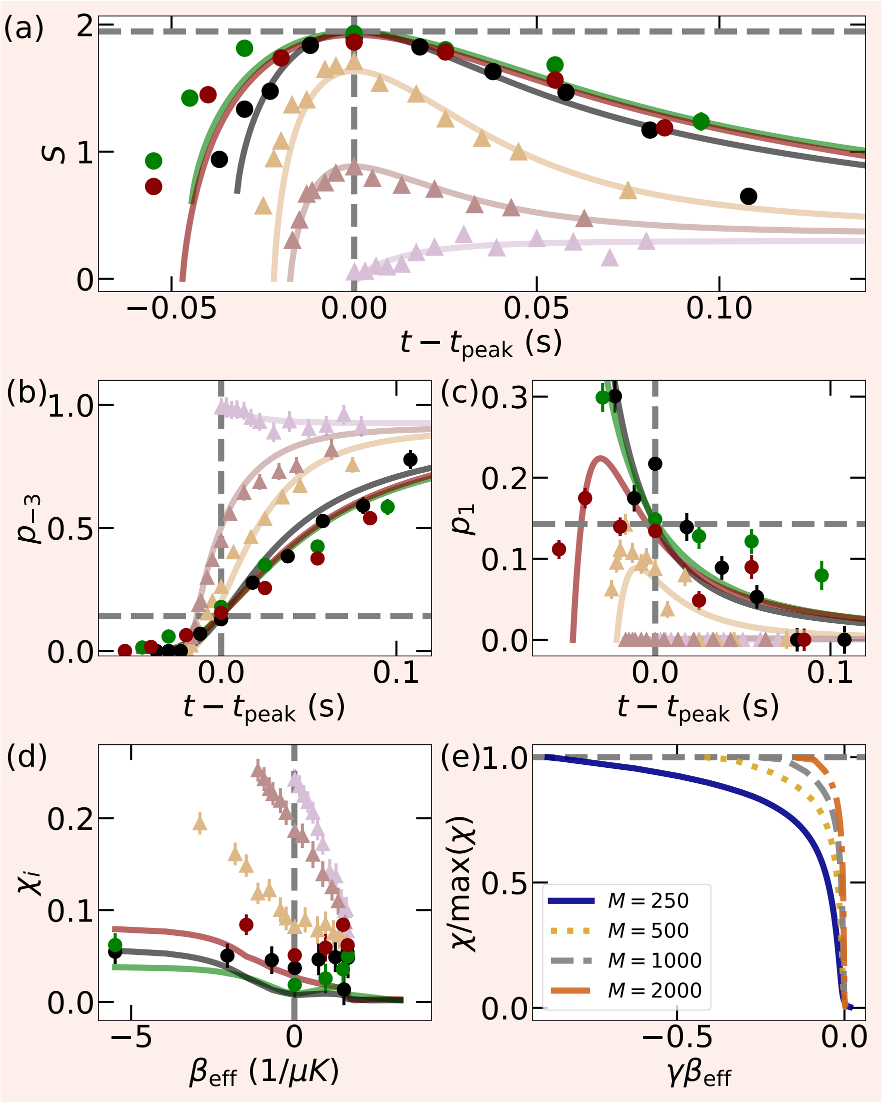

Supplementary Fig. 5. Prethermal memory loss in the bidirectional model with state-independent rates. (a) Experimentally measured (symbols) and simulated (lines) entropy for different initial conditions (color coding as in Fig. 2(b) of the main text) and (b, c) the corresponding spin population of state $|m_F\rangle$, p_{m_F}, as a function of shifted time (by the peak entropy time $t_{\rm peak}$) for the bidirectional model. As an illustration, we show two spin components. The dependency of χ (see the main text for the definition) on the control parameter $\beta_{\rm eff}$ is shown in (d) for the experiments and in (e) for the theoretical model. Horizontal grey dashed lines mark the maximal entropy $S_{\rm max}$ in (a), the population 1/7 in (b, c) that corresponds to $S_{\rm max}$, and 1 in (e). Vertical dashed lines mark $\beta_{\rm eff} = 0$. Error bars represent statistical fluctuations of 1σ standard deviation.

A. Gibbs-state ansatz

In the first approach, we assume that the system is described approximately by an effecitve Gibbs state in the vicinity of peak entropy. Note that this ansatz is not obvious (and cannot be justified by eigenstate thermalization as we deal with an open non-interacting system). However, it turns out to explain part of the behavior of the exact solution of the rate equation, like the observed critical exponents. Assuming the system to be described by a thermal state at effective inverse temperature β, the probability in the m-th eigenstate is given by

$$p_m = \frac{e^{-\beta m \Delta}}{\sum_m e^{-\beta m \Delta}} = \frac{e^{(M-m)\beta\Delta}\left(e^{\beta\Delta} - 1\right)}{e^{M\beta\Delta} - 1}. \tag{4}$$

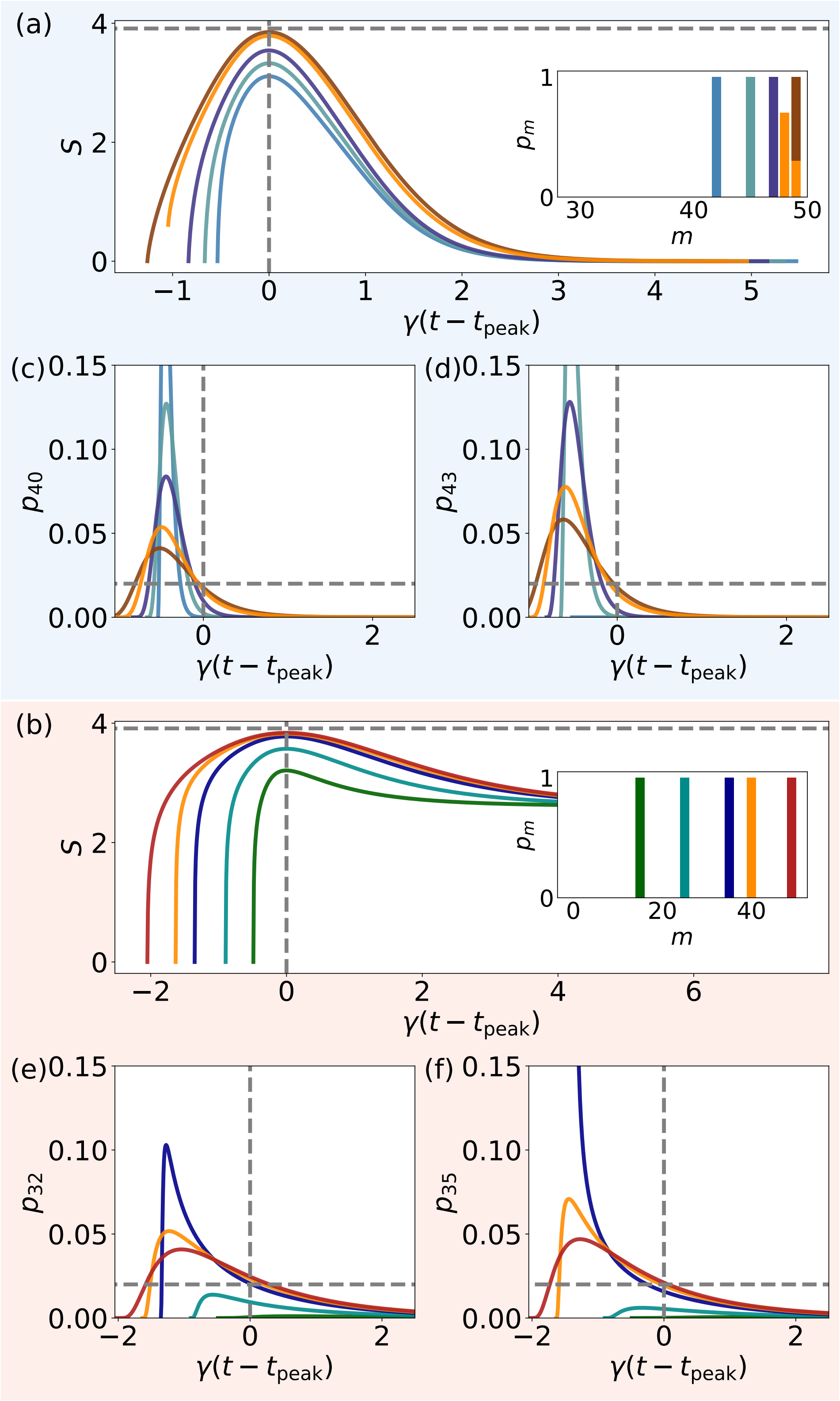

Supplementary Fig. 6. Prethermal memory loss in the theoretical models. (a, b) Entropy S and (c-f) populations on state $|m\rangle$, p_m, plotted as a function of shifted time (by the peak entropy time t_{peak}) for different initial conditions as shown in the inset. The horizontal dashed lines in (a, b) mark the maximal entropy $S_{\max} = \ln M$ with $M = 50$, in (c-f) mark $1/M = 1/50$. The vertical dashed lines mark the peak entropy time $t = t_{\mathrm{peak}}$. Background colors are the same as figures of the main text: blue for the unidirectional model, red for the bidirectional model with state-independent rates.

The corresponding localization length (participation ratio) reads

$$\xi = \left(\sum\nolimits_m p_m^2\right)^{-1} = \coth(\beta\Delta/2)\tanh(M\beta\Delta/2). \tag{5}$$

In the vicinity of $\beta = 0$, we obtain from a Taylor expansion

$$\xi/M = 1 - \frac{1}{12}(M^2-1)(\beta\Delta)^2 + \mathcal{O}(\beta^4). \tag{6}$$

For large M ($M \gg 1$), one can see that ξ is a function of $M\beta\Delta$.

We can also calculate the entropy

$$\begin{aligned} S &= -\sum\nolimits_m p_m \log p_m \\ &= \beta\Delta\left[\frac{e^{(M+1)\beta\Delta} + M - e^{\beta\Delta}(M+1)}{\left(e^{\beta\Delta}-1\right)\left(e^{M\beta\Delta}-1\right)} - M\right] \\ &\quad + \ln\frac{e^{\beta\Delta M}-1}{e^{\beta\Delta}-1}. \end{aligned} \tag{7}$$

A Taylor expansion in the vicinity of $\beta = 0$ then yields

$$\begin{aligned} S/S_{\max} &= 1 - \frac{1}{24}\frac{M^2-1}{\ln(M)}(\beta\Delta)^2 + \mathcal{O}(\beta^4) \\ &\approx 1 - \frac{1}{24}\left(\frac{M}{\sqrt{\ln(M)}}\beta\Delta\right)^2 + \mathcal{O}(\beta^4) \end{aligned} \tag{8}$$

It shows that the entropy is a function of $\beta\Delta M/\sqrt{\ln(M)}$ in the vicinity of $\beta = 0$. In Fig. 7, we plot the entropy for different system sizes as a function of $\beta\Delta$ and $\beta\Delta M/\sqrt{\ln(M)}$. Indeed, for the latter, the data are found to collapse onto each other in the vicinity of $\beta = 0$. The result from Eq. (8) is shown as the black dashed line in Fig. 7, which well describes the behavior of the entropy around the peak.

We can define an effective specific heat as $C \equiv d\langle H\rangle/dT_{\text{eff}}$, with the effective temperature $T_{\text{eff}} = 1/\beta$ and the mean energy $\langle H\rangle = \sum_m m\Delta p_m$. In the vicinity of $\beta = 0$, it can be shown that C is given by

$$C = \frac{1}{12}(\beta\Delta M)^2 + \mathcal{O}(\beta^4). \tag{9}$$

Again, we see that C is a function of $\beta\Delta M$. In Fig. 8, we show C for different system sizes as a function of $\beta\Delta$ and system-size scaled β, $\beta\Delta M$. As expected, for the latter, the data collapse onto each other in the vicinity of $\beta = 0$.

B. Continuum model

In the second approach, we map the discrete model to a continuum model. The rate equation for the discrete model reads

$$\dot{p}_m = p_{m+1}R_{-,m+1} + p_{m-1}R_{+,m-1} - p_m R_{+,m} - p_m R_{-,m}.$$

By defining

$$\begin{aligned} \nabla A_m &= (A_{m+1} - A_{m-1})/2, \\ \nabla^2 A_m &= A_{m+1} + A_{m-1} - 2A_m, \end{aligned} \tag{10}$$

one can show that

$$\begin{aligned} \dot{p}_m &= \nabla^2\left[\bar{R}_m p_m\right] + \nabla[\delta R_m p_m] \\ &\quad + \frac{1}{2}\nabla^2 p_m(\nabla\bar{R}_m + \nabla\delta R_m) + \frac{1}{2}\nabla p_m \nabla^2\delta R_m, \end{aligned} \tag{11}$$

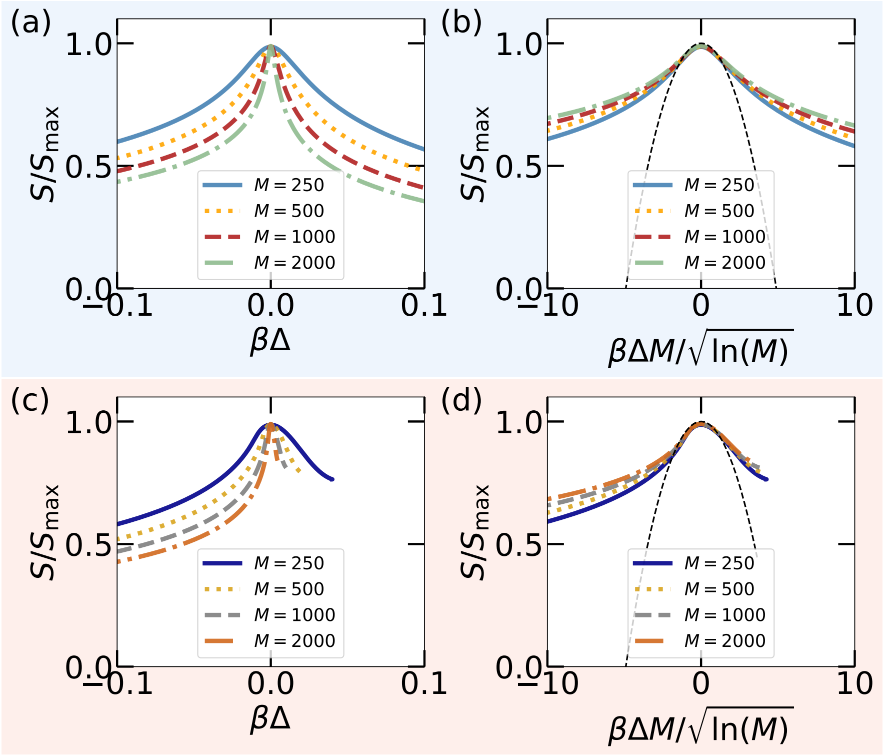

Supplementary Fig. 7. Entropy as a function of β and system-size scaled β. The solid lines are the results for the unidirectional model (a,b) and for the bidirectional model (c,d). The black dashed line is the result from Eq. (8).

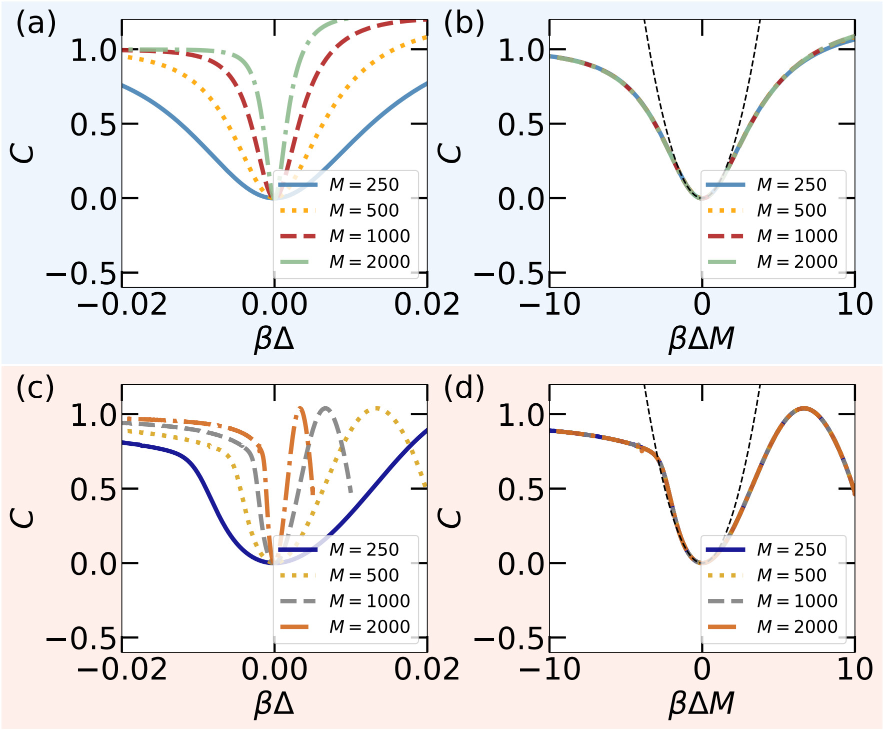

Supplementary Fig. 8. Effective specific heat $C \equiv d\langle H\rangle/dT_{\text{eff}}$ as a function of β and system-size scaled β for the unidirectional model (a,b) and for the bidirectional model (c,d). The black dashed line is the result from Eq. (9).

with

$$\bar{R}_m = (R_{+,m} + R_{-,m})/2, \quad \delta R_m = R_{-,m} - R_{+,m}.$$

Consider a system of fixed length L and coordinate $x = (m/M)L \equiv m\,\delta x$. The thermodynamic limit in the discrete model $M \to \infty$ corresponds to the limit, where x becomes continuous,

$$\delta x = \frac{L}{M} \to 0. \tag{12}$$

The corresponding rate equation for the continuum model then reads

$$\begin{aligned}\partial_t \rho(x,t) = {} & \delta x^2 \partial_x^2 \left[\bar{R}(x)\rho(x,t)\right] \\ & + \delta x \partial_x \left[\delta R(x)\rho(x,t)\right] + \mathcal{O}\left(\delta x^3\right),\end{aligned} \tag{13}$$

with $\rho(x,t)$ the probability density at x. For large M, one can neglect the high order terms $\mathcal{O}\left(\delta x^3\right)$, which brings Eq. (13) to the drift-diffusion equation,

$$\begin{aligned}\partial_t \rho(x,t) = {} & \delta x^2 \partial_x^2 \left[\bar{R}(x)\rho(x,t)\right] \\ & + \delta x \partial_x \left[\delta R(x)\rho(x,t)\right].\end{aligned} \tag{14}$$

By expanding the RHS, we obtain

$$\begin{aligned}\partial_t \rho(x,t) = {} & f(x)\rho(x,t) + g(x)\partial_x \rho(x,t) \\ & + h(x)\partial_x^2 \rho(x,t) \\ \equiv {} & A(x)\rho(x,t),\end{aligned} \tag{15}$$

where

$$\begin{aligned} f(x) &= \delta x^2 \partial_x^2 \bar{R}(x) + \delta x \partial_x \delta R(x), \\ g(x) &= 2\delta x^2 \partial_x \bar{R}(x) + \delta x \delta R(x), \\ h(x) &= \delta x^2 \bar{R}(x). \end{aligned} \tag{16}$$

The formal solution of Eq. (15) can be written as

$$\begin{aligned}\rho(x,t) &= e^{A(x)t}\rho(x,0) \\ &= [1 + A(x)t + A(x)^2 t^2/2 + \ldots]\rho(x,0),\end{aligned} \tag{17}$$

where the expansion corresponds to time-dependent perturbation theory.

Let us examine the behavior of the system starting from the maximally delocalized probability distribution, corresponding to the maximally mixed state, which approximates the state at peak entropy. Assuming $\rho(x,0) = 1/L$, corresponding to $t_{\text{peak}} = 0$, we have

$$\begin{aligned} A(x)\rho(x,0) &= f(x)/L, \\ A(x)^2\rho(x,0) &= u(x)/L, \end{aligned}$$

with

$$u(x) = f(x)^2 + g(x)\partial_x f(x) + h(x)\partial_x^2 f(x). \tag{18}$$

Plugging these expressions into the expansion (17), we obtain

$$\rho(x,t) = [1 + f(x)t + u(x)t^2/2 + \mathcal{O}(t^3)]/L, \tag{19}$$

In the following discussion, we will drop the terms $\mathcal{O}(t^3)$, which is valid for short positive and negative times t relative to the peak time.

Plugging Eq. (19) into the expression for the entropy

$$S = -\int_0^L dx \rho(x,t) \ln[\rho(x,t)\delta x], \tag{20}$$

and dropping terms of $\mathcal{O}(t^3)$, we obtain

$$\begin{aligned} S &\simeq \ln(M) \\ &\quad -\frac{1}{L}\int_0^L dx(1+ft+ut^2/2)\ln(1+ft+ut^2/2) \\ &\simeq \ln(M) \\ &\quad -\frac{1}{L}\int_0^L dx(1+ft+ut^2/2)(ft+ut^2/2-f^2t^2/2) \\ &\simeq \ln(M)-\frac{1}{L}\int_0^L dx[ft+(f^2+u)t^2/2]. \end{aligned} \tag{21}$$

The normalized entropy is then approximately given by

$$\begin{aligned} \frac{S}{S_{\max}} &\simeq 1-\frac{t}{L\ln(M)}\int_0^L dxf \\ &\quad -\frac{t^2}{2L\ln(M)}\int_0^L dx(f^2+u). \end{aligned} \tag{22}$$

The integral in the linear term is approximately equal to zero according to Eq. (19) as $\int_0^L dx\rho(x,t)=1$. Hence, the entropy reduces to

$$\frac{S}{S_{\max}} \approx 1-\frac{t^2}{2L\ln(M)}\int_0^L dx(f^2+u). \tag{23}$$

The energy is given by

$$E=\frac{\Delta}{\delta x}\int_0^L dxx\rho(x,t). \tag{24}$$

Plugging Eq. (19) into the above expression, one obtains in leading order

$$E \simeq \frac{1}{2}\Delta M + t\frac{\Delta M}{L^2}\int_0^L dxxf. \tag{25}$$

The effective inverse temperature is given by

$$\beta=\frac{dS/dt}{dE/dt}\approx -t\frac{\int_0^L dx(f^2+u)L}{\int_0^L dxxf\Delta M}. \tag{26}$$

In terms of β, the entropy reads

$$S/S_{\max} \approx 1-\frac{(\beta\Delta M)^2}{\ln(M)}\frac{(\int_0^L dxxf)^2}{2L^3\int_0^L dx(f^2+u)} \tag{27}$$

and one can see that

$$1-S/S_{\max} \propto \frac{(\beta\Delta M)^2}{\ln(M)}.$$

This is in consistent with the analytic results from Gibbs state assumption as described in the previous section.

By plugging Eq. (19) into the definition for the localization length,

$$\eta=\xi\delta x=\left[\int_0^L dx\rho(x,t)^2\right]^{-1}, \tag{28}$$

we find

$$\eta^{-1} \approx \frac{1}{L}+\frac{1}{L^2}\int_0^L dx(f^2+u)t^2. \tag{29}$$

In the finite-size scaling analysis for the unidirectional model with time as control parameter, the overall strength of the bath-induced rates scales with the system size as $M^{1.16}$, in which case the entropy reaches peak around the same time for different system sizes. Therefore, $\sqrt{f^2+u} \propto \delta x M^{1.16} \propto M^{0.16}$. It indicates that the localization length is a function of $M^{0.16}t$, which is consistent with the numerical results as shown in Fig. 4.

In terms of β, the localization length reads

$$\begin{aligned} \eta/L &= \xi/M \\ &\approx \left\{ 1 + (\beta\Delta M)^2 \frac{(\int_0^L dx x f)^2}{L^3 \int_0^L dx (f^2+u)} \right\}^{-1} \\ &\approx 1 - (\beta\Delta M)^2 \frac{(\int_0^L dx x f)^2}{L^3 \int_0^L dx (f^2+u)}. \end{aligned} \tag{30}$$

Hence, ξ/M is a function of $\beta\Delta M$. Similar analysis shows that the effective specific heat is also a function of $\beta\Delta M$.

SUPPLEMENTARY REFERENCES

[1] F. Schmidt, D. Mayer, Q. Bouton, D. Adam, T. Lausch, J. Nettersheim, E. Tiemann, and A. Widera, Tailored single-atom collisions at ultralow energies, Phys. Rev. Lett. **122**, 013401 (2019).

[2] M. Cannoni, Relativistic $\langle \sigma v_{\rm rel} \rangle$ in the calculation of relics abundances: A closer look, Phys. Rev. D **89**, 103533 (2014).

[3] T.-L. Ho, Spinor bose condensates in optical traps, Phys. Rev. Lett. **81**, 742 (1998).

[4] Q. Bouton, J. Nettersheim, D. Adam, F. Schmidt, D. Mayer, T. Lausch, E. Tiemann, and A. Widera, Single-atom quantum probes for ultracold gases boosted by nonequilibrium spin dynamics, Phys. Rev. X **10**, 011018 (2020).
